# Anthropometric prediction of DXA-measured body composition in female team handball players

**DOI:** 10.7717/peerj.5913

**Published:** 2018-11-27

**Authors:** Valentina Cavedon, Carlo Zancanaro, Chiara Milanese

**Affiliations:** Laboratory of Anthropometry and Body Composition, Department of Neurosciences, Biomedicine and Movement Sciences, University of Verona, Verona, Italy

**Keywords:** Handball players, Anthropometry, Fat mass, Skeletal muscle, Skinfolds, Predictive equation

## Abstract

**Background:**

The relevance of body composition (BC) to performance in sport has long been appreciated with special concern on the total and regional proportion of fat and muscle. Dual-energy X-ray absorptiometry (DXA) is able to accurately measure BC, but it may not be easily available in practice; anthropometry has long been used as a simple and inexpensive field method to objectively assess BC. The aim of this study was twofold: first, to develop and validate a sport-specific anthropometric predictive equation for total body fat mass (FM) and lean mass components in female handball players to be used in the sport setting; second, to cross-validate in female team handball players several independently developed, predictive equations for BC in female athletes.

**Methods:**

A total of 85 female team handball players (30 wings, 31 backs, 14 pivots, 10 goalkeepers) of different competitive levels underwent anthropometry and a whole-body DXA scan. Multiple linear regression analysis was used to develop predictive equations in a derivation sample (*n* = 60) of randomly selected players using demographic and anthropometric variables. The developed equations were used to predict DXA outcomes in an independent validation sample (*n* = 25).

**Results:**

Statistically significant (*P* < 0.001) models were developed for total body FM (adjusted *R*^2^ = 0.943, standard error of the estimate, SEE = 1,379 g), percentage FM (adjusted *R*^2^ = 0.877, SEE = 2.00%), fat-free soft tissue mass (FFSTM) (adjusted *R*^2^ = 0.834, SEE = 2,412 g), fat-free mass (FFSTM + bone mineral content; adjusted *R*^2^ = 0.829, SEE = 2,579 g). All models were robust to collinearity. Each developed equation was successfully validated in the remaining 25 players using correlation analysis, mean signed difference, *t*-test, and Bland–Altman plot. The whole dataset of team handball players (*n* = 85) was used to cross-validate several predictive equations independently developed by others in female athletes. Equations significantly (*P* < 0.001 for all; *t*-test) over- or underestimated the corresponding DXA measurements.

**Discussion:**

It is concluded that in team female handball players the anthropometric equations presented herein are able to estimate body fat and FFSTM with accuracy. Several BC predictive anthropometric equations developed in different female athletic populations revealed inaccurate when tested in team handball players. These results should be of use for coaches, physical trainers, and nutritionists when evaluating the physical status of female team handball players.

## Introduction

Team handball (hereinafter, handball) is a popular team sport practiced worldwide by about 18 million players in more than 150 international federations (Marques et al., 2007; Raeder, Fernandez-Fernandez & Ferrauti, 2015). Handball is a high-intensity sport and one of the most physically demanding sport games consisting of intense, intermittent movements such as running, jumping, sprinting, throwing, hitting, blocking, and pushing (Gorostiaga et al., 2006). Handball also demands strength and power in both the upper and the lower limb muscles (Chelly, Hermassi & Shephard, 2010).

The evaluation of body composition (BC) is a key issue in sports science as well as sports practice with special reference to the body content of fat and skeletal muscle. The total and regional BC is related to performance (Leedy et al., 1965; Behnke & Royce, 1966; Stewart, 2001) as well as to the risk of injury (Duthie, 2006). Indeed, it has been shown (Reilly, 1996) that fat mass (FM) acts as a dead weight to be lifted against gravity during activities such as jumping and sprinting and also affects energy expenditure; moreover, FM is inversely related to aerobic capacity, players’ power-to-weight ratio, and thermoregulation (Gabbett, 2005). In contrast, skeletal muscle mass, that is, the main component of body fat-free mass (FFM), strongly contributes to strength and power performance. Accordingly, monitoring changes in body FM or skeletal muscle mass during training, in-season or off-season is of utmost interest to athletes, coaches, and physical trainers because of the relationships that body adiposity and lean mass share with performance, and the need for monitoring the effects of training, coaching, and competition (Albanese, Diessel & Genant, 2003; Hoshikawa et al., 2005; Duthie, 2006; Carling & Orhant, 2010; Milanese et al., 2015).

Several different methods and tools are available to assess BC with these methods ranging in costs, practicality and accuracy. Dual-energy X-ray absorptiometry (DXA) is currently considered the gold standard in accurately measuring BC in terms of FM, fat-free soft tissue mass (FFSTM), bone mineral content (BMC), and FFM (sum of FFSTM and BMC) (Devlin et al., 2017; Prioreschi et al., 2018) and its practice is spread among athletic populations (Ackland et al., 2012). Unfortunately, DXA may not be easily available in practice due to logistic and cost reasons. Anthropometry has long been used as a simple and inexpensive field method to objectively assess BC (Tran & Weltman, 1988, 1989) and it is also considered a robust method of assessment of BC in athletic populations (Reilly et al., 2009; Ackland et al., 2012). A number of different regression equations have been developed in different populations in order to estimate FM and percentage FM (%FM) as well as FFM and skeletal muscle mass from body measurements, possibly in combination with age (Madden & Smith, 2016; Cortés-Castell et al., 2017). However, the use of a predictive equation is advocated only if being applied to the population from which it was derived (Reilly et al., 2009). The application of generic predictive equations for assessing BC in a particular athletic population may cause errors due to the distinct fat patterning that is specific for each sport (Reilly et al., 2009). Sport-specific predictive equations have been developed against a gold standard in male soccer players (Reilly et al., 2009) and in male rugby players (Zemski, Broad & Slater, 2018). DXA-validated predictive equations have been previously developed on different female athletic populations (Fornetti et al., 1999; Warner et al., 2004; Evans et al., 2005; Santos et al., 2015). However, the above quoted predictive equation for the female athletic population (Fornetti et al., 1999; Warner et al., 2004; Evans et al., 2005; Santos et al., 2015) were developed on samples of athletes practicing several (up to 15) different sports.

Despite the worldwide diffusion of handball, to the best of our knowledge there are no handball-specific anthropometric equations available to predict BC in handball players. Preliminary work conducted in our laboratory on a small sample of female handball players showed that available predictive equations developed using DXA in athletic populations are not accurate in predicting BC in handball participants. Therefore, in the current study we recruited a number of female handball players to develop and validate sport-specific anthropometric predictive equations for body fat and lean mass components using DXA as the criterion. Further, we cross-validated in female handball players several independently DXA-developed predictive equations for %FM or FFM in female athletes (Fornetti et al., 1999; Warner et al., 2004; Evans et al., 2005; Santos et al., 2015). The observations of the current study would be of great importance in this body of literature by adding relevant data about sport-specific predictive equations and filling the knowledge gap in this specific athletic population of handball players. Finally, if demonstrated accurate, the DXA-derived equations in the current study may offer a practical and inexpensive tool for coaches and physical trainers for assessing BC in female handball players.

## Methods

### Participants

A priori analysis conducted with G-Power (Faul et al., 2009) showed that, assuming the proportion of variance explained by an individual predictor to be 15% (*f*^2^ = 0.15) (Cohen, 1988) in multiple regression analysis (two tails; power, 0.90; α = 0.050; number of predictors = 8) a sample size of 73 participants was required. Taking into account a ≈20% dropout, 87 participants were initially recruited. The Institutional Review Board of the University of Verona approved the study protocol (prot. 11597/09.11.01), which was in accordance with the Helsinki Declaration. All participants signed informed consent.

Participants were playing in six different teams participating in the following Italian national championships ordered from the highest to lowest competitive level: A1 (*n* = 32), A2 (*n* = 38), B (*n* = 17). According to playing position, the participants encompassed 30 wings, 32 backs, 15 pivots, 10 goalkeepers. All measurements were taken at the beginning of the competitive season in October. During pre-season, A1 players had been training an average of eight sessions a week, 3.5 h per session; A2 and B players had been training an average of three sessions a week, 2.0 h per session. All participants had at least 1 year of competitive handball experience. At the time of measurements all participants were in good health and have had no major injury in the last 6 months.

### Measurements

Measurements were taken on the same day in the morning after a 3–4 h fast. All participants were invited to void their bladder and evacuate before measurement. Participants with menses were invited to postpone the measurement session by at least 8 days. All participants were instructed to avoid strenuous exercise in the 24 h preceding measurement. Body mass was taken at the nearest 0.1 kg with an electronic scale (Tanita electronic scale BWB-800 MA) and stature was measured with a Harpenden stadiometer (Holtain Ltd, Crymych, Pembs, UK) at the nearest 0.01 m, according to the International Society for Advancement of Kinanthropometry (2001). Both measurements were taken with the subject wearing no shoes and minimum clothing. Body mass index (BMI) was calculated as weight (kg)/height (m)^2^. The following lengths (L) and breadths (B) were measured with a Harpenden anthropometer (Holtain Ltd, Crymych, Pembs, UK): shoulder-elbow L, elbow-wrist L, thigh L, tibialelaterale to floor L, transverse chest B, anterior–posterior (A–P) chest B, elbow B, wrist B, knee B, and ankle B. Body circumferences (C) were measured with a fiberglass tape at the upper arm (relaxed), waist, hip, thigh, and calf sites. Skinfold (SF) thickness was measured in duplicate with a Harpenden calliper (Gima, Milan, Italy) at the triceps, axillary, subscapular, suprailiac, abdominal, anterior thigh, and calf site. The average of the two readings was the measure. If the two measures differed by more than two mm, a third measurement was taken, and the two closest were then averaged and recorded as the score. All measurements were taken by an experienced anthropometrist according to standard procedures (Lohman, Roche & Martorell, 1988; Norton & Olds, 1996).

Fat mass (g), %FM, FFSTM (g), BMC (g), and FFM (g) were measured using a DXA scanner (QDR Explorer W; Hologic, MA, USA; fan-bean technology, software for Windows XP version 12.6.1), according to the manufacturer’s procedures. To avoid possible baseline drift, the scanner was checked daily against a standard anthropomorphic spine phantom supplied by the manufacturer. All scans were performed by one operator, in order to ensure consistency. In our lab the precision error (percent coefficient of variation with repositioning) of whole-body DXA measurements is 1.1%, 2.3%, 0.5%, and 2.8% for BMC, FM, FFSTM, and %FM, respectively. All participants were asked about possible pregnancy prior to DXA scan measurements. In the case of possible pregnancy the participant was excluded from the study. During scanning, participants wore lightweight clothing with no metal or reflective material, and removed all metal accessories.

A total of 85 participants completed all measurements (30 wings, 31 backs, 14 pivots, 10 goalkeepers) and were used in analysis.

### Statistical analysis

The test-retest reliability of the anthropometric measurements was assessed with the intraclass correlation coefficient (ICC 3,1) with a two-way mixed single measures model and absolute agreement. For regression analysis, the 85 participants were randomly assigned to a derivation sample (*n* = 60) and a validation sample (*n* = 25). Continuous variables in the two groups were compared with the Student’s *t*-test for paired samples. The equality of proportion of players in the two groups according to the competitive level (i.e., A1, A2, and B) or the playing position (i.e., W, B, P, and GK) was assessed with the Pearson Chi-squared test. Correlation between variables was assessed with the Pearson’s correlation coefficient *r*. Sport-specific predictive equations were developed in the derivation sample by running backward multiple regression analyses with DXA-measured BC outcomes (FM, %FM, FFSTM, FFM) as the dependent variable and anthropometric and demographic variables as the predictors. Predictor variables were selected up to a maximum of eight using both of the following criteria: (i) higher significant correlation (*r*) with the dependent variable and (ii) *r* value >0.200. The probability of F-to-enter was set at ≤0.05 for inclusion and ≥0.10 for exclusion of predictor variables. Adjusted *R*^2^ and standard error of the estimate (SEE) were used to assess the goodness-of-fit of the prediction model. Homoscedasticity of data was assessed by both plotting the residuals of multiple regression analysis against the predicted values and the Koenker test (Koenker, 1981). The presence of serial correlations among the residuals was tested using the Durbin–Watson statistic; the variance inflation factor was calculated to check for multicollinearity in the multiple linear regression models. The effect size (Cohen’s *f*^2^), (Cohen, 1988) for a given predictor (p) with all others left in the model was calculated according to the formula: $f_p^2 = {\rm{ }}{R^2}-R_{k-1}^2/1-{R^2}$, where *R*^2^ is the coefficient of determination and *R*_k−1_^2^ is the coefficient of determination in the absence of *p*. According to Cohen’s guidelines (Cohen, 1988), effect size values were interpreted as small (*f*^2^ = 0.02), medium (*f*^2^ = 0.15) and large (*f*^2^ = 0.35). The developed equations were used to predict DXA outcomes in the validation sample. The validity of the developed equations was evaluated with the correlation coefficient *r*, the coefficient of determination (*R*^2^), the Student’s *t*-test, the mean signed difference (MSD) and the Bland–Altman plot. The same procedure was used to cross-validate against DXA measurements several BC predictive equation independently developed in female athletes (Table 1). Statistical analyses were performed using SPSS v. 22 (IBM Corp., Armonk, NY, USA). Post hoc statistical power was evaluated using G*Power Software 3.1 (Faul et al., 2009) on the basis of the sample size and the observed effect sizes. The alpha value was set at 0.05.

**Table 1:** Predictive equations previously developed in various types of female athlete populations.

Reference	Predicted variable	Predictor variable	Constant	B coefficients
Santos et al. (2015)	FM (%)	Age (y), stature (cm), hip C (cm), waist C (cm)	−7.74	−0.32, −0.26, 0.54, 0.33
Evans et al. (2005)		Sum of the triceps, suprailiac, and thigh SF (mm), sex, race Sex, *F* = 0; Race, White = 0	8.977	0.24658, −6.343, −1.998
Warner et al. (2004)	FFM (kg)	Weight (kg), abdominal SF (mm), thigh SF (mm)	8.51	0.809, −0.178, −0.225
Fornetti et al. (1999)		Height (cm), weight (kg)	−10.03	0.143, 0.565

**Note:**

%FM, percent fat mass; C, limb girth at the specific site; SF, skinfold; FFM, fat-free mass (FFSTM + bone mineral content).

## Results

### Participants’ characteristics

The characteristics of female handball players in the whole group of participants (*n* = 85) as well as the derivation (*n* = 60) and validation (*n* = 25) samples are summarized in Table 2. The derivation and validation samples were similar for demographic and BC variables (Student’s *t*-test) as well as distribution across competitive levels and playing positions (Pearson Chi-squared test) (Table 2).

**Table 2:** Characteristics of the participant female handball players in the whole group (W), the derivation group (D) for predictive equation, and the validation group (V).

Variable	W (*n* = 85)	D (*n* = 60)	V (*n* = 25)	P
Age (y)	22.1 ± 5.7	21.8 ± 5.6	22.7 ± 5.9	0.528
Body mass (kg)	64.9 ± 10.7	65.1 ± 11.4	64.5 ± 8.8	0.808
Stature (cm)	167.4 ± 6.2	167.7 ± 6.2	166.5 ± 6.4	0.401
BMI (kg/m^2^)	23.1 ± 3.1	23.0 ± 3.3	23.2 ± 2.9	0.806
Triceps SF (mm)	16.3 ± 4.4	16.3 ± 4.2	16.2 ± 4.9	0.994
Subscapular SF (mm)	12.6 ± 5.7	12.8 ± 5.8	12.3 ± 5.4	0.696
Thorax SF (mm)	7.4 ± 2.3	7.4 ± 2.4	7.4 ± 2.0	0.996
Axillary SF (mm)	10.8 ± 5.7	10.75 ± 5.9	10.8 ± 5.8	0.981
Abdominal SF (mm)	20.2 ± 6.7	20.5 ± 7.3	19.6 ± 5.4	0.605
Suprailiac SF (mm)	16.1 ± 6.9	16.3 ± 7.4	15.7 ± 5.6	0.735
Thigh SF (mm)	22.5 ± 6.0	22.7 ± 6.0	22.1 ± 5.8	0.653
Calf SF (mm)	14.2 ± 4.4	14.4 ± 4.6	13.6 ± 3.6	0.440
Sum of SFs (mm)	120.2 ± 33.7	121.2 ± 43.8	117.8 ± 31.5	0.680
Arm C (cm)	27.4 ± 2.5	27.4 ± 2.6	27.4 ± 2.3	0.970
Wrist C (cm)	15.5 ± 0.8	15.6 ± 0.8	15.4 ± 0.7	0.432
Waist C (cm)	73.4 ± 6.9	73.6 ± 7.5	73.1 ± 5.2	0.778
Hip C (cm)	100.1 ± 7.5	101.1 ± 7.9	100.6 ± 6.4	0.753
Thigh C (cm)	52.5 ± 4.9	52.3 ± 5.1	53.1 ± 4.1	0.491
Calf C (cm)	37.5 ± 3.9	37.6 ± 4.4	37.2 ± 2.2	0.664
S-H L (cm)	30.5 ± 1.6	35.6 ± 1.6	35.4 ± 1.6	0.620
Elbow–wrist L (cm)	26.8 ± 1.5	26.8 ± 1.6	26.8 ± 1.30	0.978
Thigh L (cm)	39.4 ± 2.3	39.5 ± 2.4	39.3 ± 2.3	0.796
Tl-to-floor L (cm)	45.0 ± 2.6	44.9 ± 2.6	45.0 ± 2.6	0.922
Tho Tr B (cm)	26.5 ± 2.6	26.6 ± 2.7	26.1 ± 2.5	0.360
Tho A–P B (cm)	18.8 ± 2.1	18.7 ± 1.9	18.9 ± 2.9	0.705
Elbow B (cm)	6.2 ± 0.4	6.2 ± 0.4	6.3 ± 0.4	0.390
Wrist B (cm)	5.1 ± 0.3	5.1 ± 0.3	5.1 ± 0.2	0.870
Knee B (cm)	9.7 ± 0.7	9.7 ± 0.7	9.7 ± 0.6	0.782
Ankle B (cm)	6.6 ± 0.5	6.6 ± 0.5	6.6 ± 0.4	0.896
FM (g)	16673.7 ± 6186.3	16808.9 ± 6567.7	16348.9 ± 5268.5	0.975
%FM	25.4 ± 5.6	25.4 ± 5.9	25.2 ± 5.1	0.855
FFSTM (g)	45202.1 ± 5706.5	45254.3 ± 6011.5	45076.8 ± 5011.2	0.897
FFM (g)	47635.8 ± 6013.4	47686.8 ± 6340.5	47513.2 ± 5265.7	0.863
CL (A1/A2/B)	31/38/16	24/25/11	7/13/5	0.564
PP (W/B/P/GK)	30/31/14/10	23/23/9/5	7/8/5/5	0.386

**Notes:**

*P*-value of ANOVA or Chi-squared test for D vs. V. Data are mean ± SD.

BMI, body mass index; SF, skinfold; C, limb girth at the specific site; L, length; B, breadth; A–P, anterior–posterior; Tl-to-floor, tibialelaterale-to-floor; FM, fat mass; %FM, percent fat mass; FFSTM, fat-free soft tissue mass; FFM, fat-free mass (FFSTM + bone mineral content); CL, competitive level; A, A1, B, competitive level in Italian championships; PP, playing position; W, wing; B, back; P, pivot; GK, goalkeeper.

### Regression analysis and validation of prediction equations

The test-retest ICC (3,1–single measures) with 95% confidence interval (CI) for the anthropometric measurements was 0.993 (95% CI [0.946–1.000]) indicating excellent reliability (Portney & Watkins, 2000).

**Figure 1:**
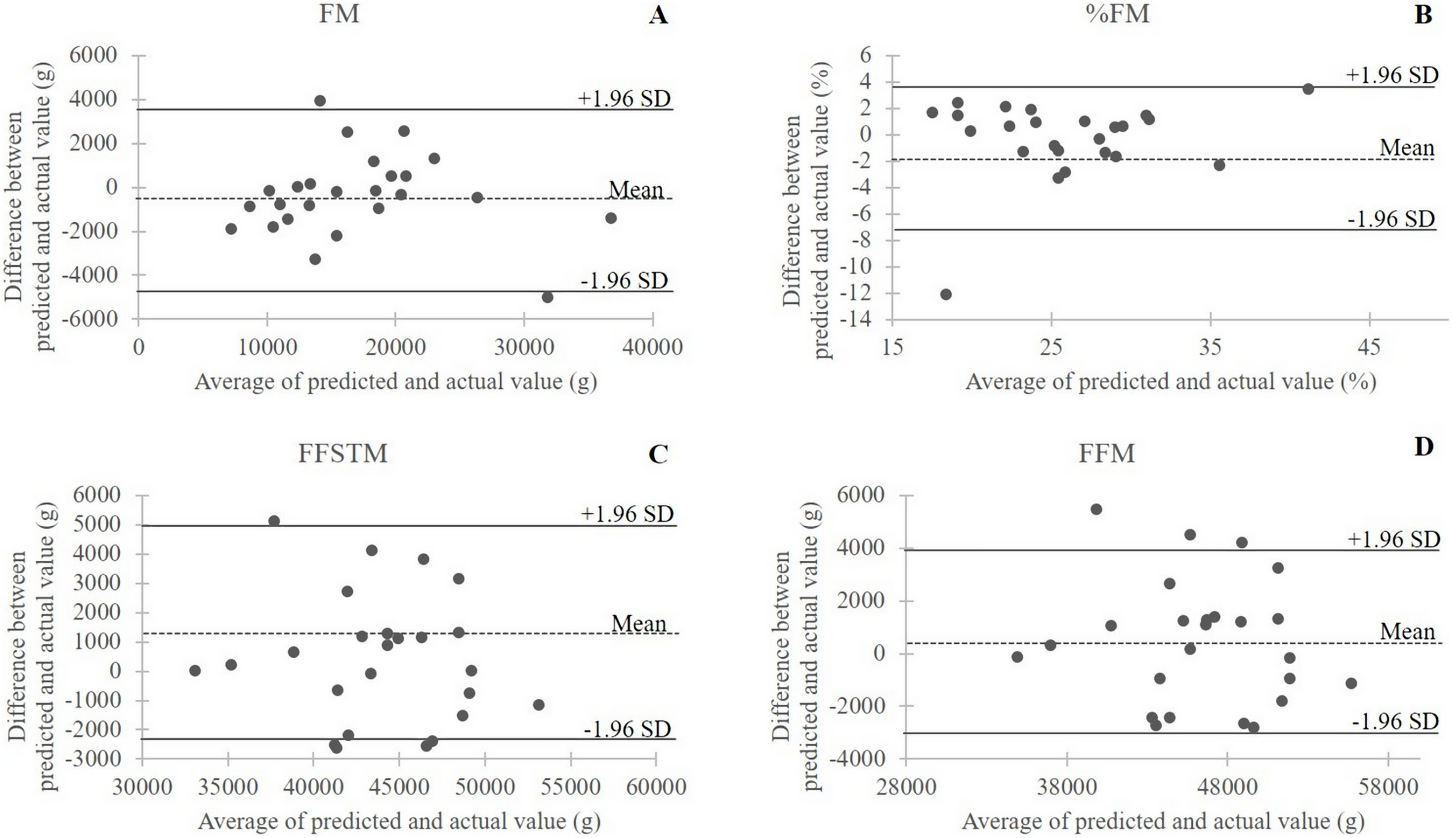
Bland–Altman plots of the differences between actual (DXA) measurements and predicted values in the validation sample (*n* = 25) of female handball players. The dotted line is the average of the differences; the bold lines are the 95% limits of agreement. (A) FM, body fat mass; (B) %FM, percent of body fat mass; (C) FFSTM, body fat-free soft tissue mass; (D) FFM, fat-free mass.

Results of regression analysis in the derivation sample (*n* = 60) are presented in Table 3. All of the developed models were statistically significant (*P* < 0.001). Adjusted *R*^2^ ranged from 0.798 (FFM) to 0.943 (FM). For all models, the Durbin–Watson statistics was between 1.5 and 2.5 and the variance inflation factor was <5.000 for each predictor, indicating robustness to collinearity. The Koenker test for homoscedasticity of data was not significant (*P* > 0.05) for all models. Post hoc power analyses revealed that the statistical power for regression analyses in this study exceeded 0.99, suggesting good model sensitivity to Type II error as well as that the models were adequately powered to detect the true effect of the predictor variables. In the validation sample, (*n* = 25) the correlation between DXA-measured and predicted BC variables was statistically significant for all of the developed equations (r value ranging from 0.870 to 0.996; *P* < 0.001 for all; post hoc statistical power >0.99) and no significant difference was found on *t*-test (Table 4); MSD showed that all the developed predictive equations slightly overestimated the criterion value apart from FM and %FM. In Bland–Altman plots (Fig. 1) at least 92% of the data points in each plot fell within the 95% limits of agreement.

**Table 3:** Predictive equations developed in the derivation sample of female handball players (*n* = 60).

Predicted variable	Predictor variable	Adj *R*^2^	Constant	B coefficient	*f*^2^	SEE
FM (g)	S8SF (mm), hip C (cm), thorax A–P B (cm)	0.943	−36300.25	101.46, 364.63, 206.82	16.51	1379.64
FM (%)	S8SF (mm), hip C (cm), arm C (cm), Waist C (cm), calf C (cm)	0.877	10.01	0.17, 0.30, −0.44, −0.17, −0.29	7.13	2.00
FFSTM (g)	BM (kg), elbow B (cm), stature (cm)	0.834	−49295.84	271.79, 2123.80, 380.84	5.02	2412.25
FFM (g)	BM (kg), elbow B (cm), stature (cm)	0.829	−53156.59	278.27, 2294.04, 409.64	4.85	2579.32

**Note:**

FM, fat mass; %FM, percent fat mass; FFSTM, fat-free soft tissue mass; FFM, fat-free mass (FFSTM + bone mineral content); SF, skinfold; S8SF, sum of 8 skinfolds; A–P, anterior–posterior; B, breadth; C, limb girth at the specific site; BM, body mass; Adj *R*^2^, adjusted *R*^2^; SEE, standard error of estimate.

**Table 4:** Comparison between predicted and measured body composition variables in the validation sample of female handball players (*n* = 25).

Variable	Correlation analysis			Paired *t*-test		MSD	95%CIs for MSD	LOA (B-A)
	*r*	*P*-value	*R*^2^	*t*	*P*-value			
FM (g)	0.996	<0.001	0.932	0.722	0.477	−269.2	−1038.9, +500.5	3385.8, −3924.2
%FM	0.870	<0.001	0.757	0.368	0.716	−0.2	−1.5, +1.0	5.7, −6.1
FFSTM (g)	0.897	<0.001	0.805	1.113	0.277	485.5	−415.0, +1386.1	4761.7, −3790.6
FFM (g)	0.894	<0.001	0.798	1.108	0.279	518.7	−447.4, +1484.9	5106.2, −4068.8

**Note:**

FM, fat mass; %FM, percent fat mass; FFSTM, fat-free soft tissue mass; FFM, fat-free mass (FFSTM + bone mineral content); MSD, mean signed difference; CIs, confidence intervals; LOA (B–A), Limits of agreement in Bland–Altman analysis.

### Cross validation of anthropometric equations

**Figure 2:**
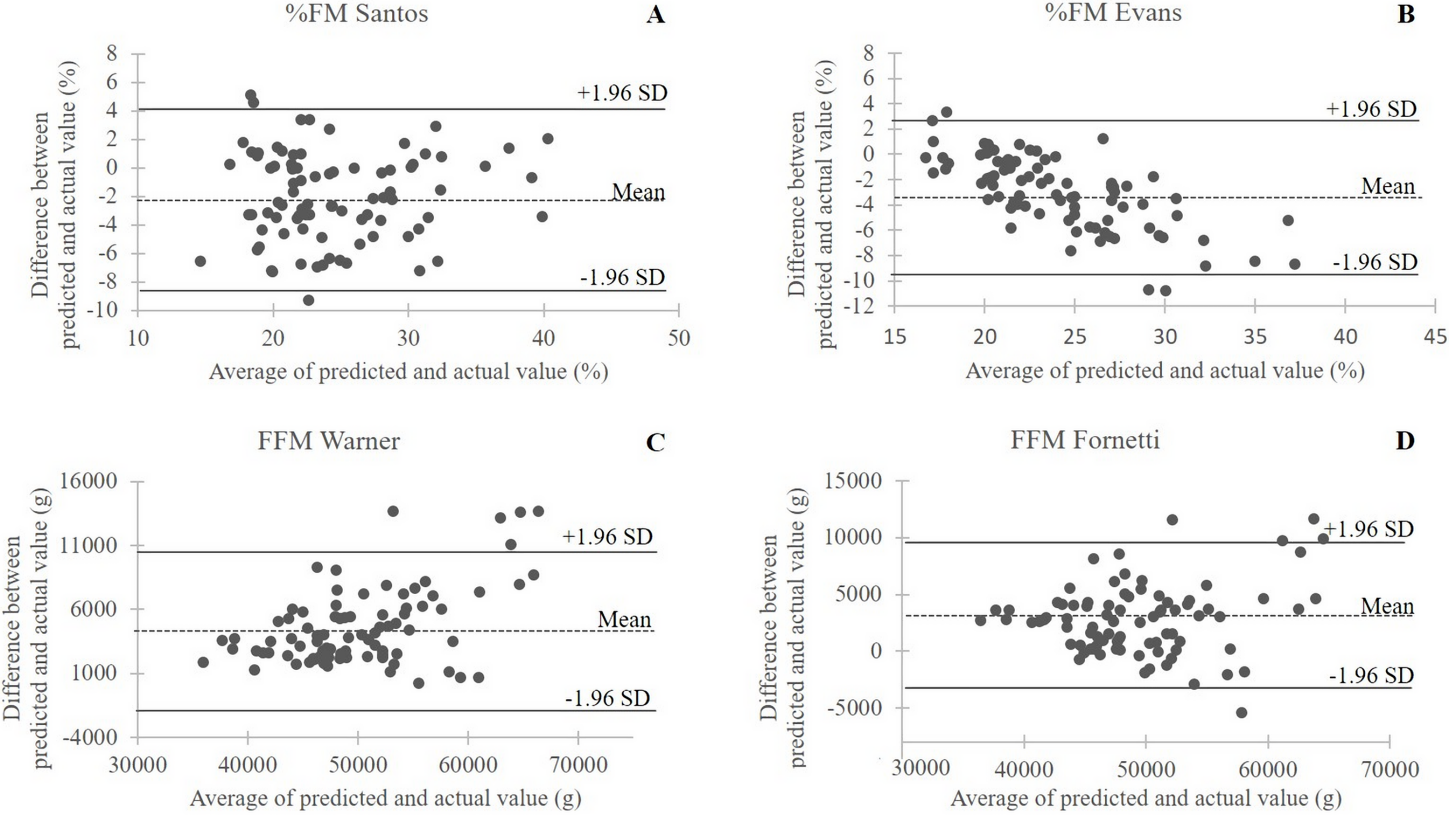
Bland–Altman plots of the differences between actual (DXA) measurements and predicted values in the validation whole sample (*n* = 85) of female handball players. The dotted line is the average of the differences; the bold lines are the 95% limits of agreement. (A) %FM Santos, %FM predicted with the equation of Santos et al. (2015); (B) %FM Evans, %FM predicted with the equation of Evans et al. (2005); (C) FFM Warner, fat-free mass predicted with the equation of Warner et al. (2004); (D) FFM Fornetti, fat-free mass predicted with the equation of Fornetti et al. (1999).

Body composition variables estimated with anthropometric equations specifically developed in female athletes (Table 1) and DXA outcomes measured in 85 female handball players were highly correlated (*r*, 0.850–0.923; *P* < 0.001 for all). However, the Student’s *t*-test showed a systematic error between predicted BC variables and the corresponding DXA measurements for all equations (*P* < 0.001 for all; post hoc power >0.99 for all). In particular, the equation of Santos et al. (2015) and Evans et al. (2005) underestimated %FM vs. DXA (23.4 ± 5.79 vs. 25.4 ± 5.61 with MSD = −1.89%; 22.5 ± 5.79 vs. 25.4 ± 5.61 with MSD = −2.83, respectively) and the equations of Warner et al. (2004) and Fornetti et al. (1999) overestimated FFM vs. DXA (52,359 ± 7,491 vs. 47,635 ± 6,013 g, MSD = +4,723 g; 50,581 ± 6,580 vs. 47,635 ± 6,013 g; MSD = +2,945 g). The Bland–Altman plot (Fig. 2) showed good agreement between actual and estimated values with a very limited number of outliers and at least 94.2% of the data points in each plot falling within the 95% limits of agreement.

## Discussion

Evaluating and monitoring BC is a key issue in sports practice due to its link to performance and injury risk prevention. The results of this study offer a novel, handball-specific, DXA validated tool for estimating BC in female handball players using anthropometry, thereby filling a knowledge gap in the literature. Moreover, results showed that DXA-developed BC predictive equations obtained on different female athletic populations (Fornetti et al., 1999; Warner et al., 2004; Evans et al., 2005; Santos et al., 2015) might be inaccurate in handball players. This further underlines the need for sport-specific predictive equations for estimating BC in athletic populations.

A first result of this study was that anthropometric equations are able to predict FM and %FM in female handball players with accuracy (percentage of in-sample explained variance = 94.3 and 87.7; SEE = 1.4 kg and 2%, respectively). Validation of the two developed equations in a different sample of female handball players showed excellent agreement between predicted and measured FM and %FM values. Two anthropometric predictors namely, sum of SF and hip C, showed the largest effect size in predicting FM and %FM (*f*_ΣSF_^2^ = 9.53 and 7.84; *f*_hipC_^2^ = 7.09 and 2.25, respectively). This was unsurprising, because both anthropometric variables are closely associated with total body adiposity in female athletes (Mayhew et al., 1983; Santos et al., 2014). FFSTM, that is, FFM exclusive of bone mineral, and FFM, that is, the sum of FFSTM and BMC were predicted with accuracy (percentage of explained variance >80%, SEE ≈ 2.5 kg for both) by body size, that is, body mass and stature: *f*_BM_^2^ = 2.69 and 2.54; *f*_stature_^2^ = 2.03 and 2.03, respectively. This is in accordance with lean mass representing the large majority of body mass in normal weight subjects. Taken together, these findings indicate that the anthropometric equations developed in the current study accurately estimate DXA-measured FM and FFM and can therefore be used as an accurate field tool to assess body fat and lean mass in female handball players.

The second objective of this study was to examine the cross-validation in handball players of DXA-derived predictive equations for %FM or FFM previously developed in female athletes. Observations revealed that while highly significant correlations between measured and predicted values were found for all equations indicating satisfactory agreement, the *t*-test revealed a significant systematic error for all equations leading to under- or overestimation of DXA-measured BC variables in handball players.

First, the equation of Santos et al. (2015) predicting %FM was developed in 50 elite athletes of unknown ethnicity practicing diverse sport activities (inclusive of handball, *n* = 4) with a four-compartment model as the criterion; the predictor variables were age, stature, and hip and waist C with a predicted residual error sum of squares (PRESS) *R*^2^ of 0.52 and PRESS SEE of 3.35%. In female handball players, this equation significantly (*P* < 0.001) underestimated %FM with a moderate MSD of −1.89%. This discrepancy may be due to the limited number of participating athletes in the study of Santos et al. (2015) possibly leading to the moderate coefficient of determination and the relatively large SEE. Moreover, the diversity of practiced sports and the absence of SF in the set of predictors selected may have affected the agreement with the sport-specific predictive equation developed in our sample of handball players. The next equation used for cross-validation was that of Evans et al. (2005), which was developed using a four-compartment model as the criterion in a multi-ethnic group of male and female collegiate athletes practicing football, basketball, volleyball, gymnastics, swimming, and track and field. The predictors were sum of 7 SF, gender, and race. When applied in female handball players, this equation significantly (*P* < 0.001) underestimated %FM vs. DXA measurement (MSD = −2.83). However, both female (mean %FM = 18.7) and male (mean %FM = 11.7) participants in the study of Evans et al. (2005) had lower levels of adiposity than our handball players (mean %FM = 25.4); this may affect the performance of the equation where SFs are essential predictors. Another equation used in this study for cross-validation was that of Warner et al. (2004), which was developed in 101 National Collegiate Athletic Association Division I athletes (none practicing handball), using DXA as the criterion. The participants had similar body mass (63.1 ± 8.1 kg), stature (166.7 ± 7.8 cm), and BMI (22.6 ± 2.0 kg/m^2^) as the sample of handball players participating in the current study. Predictors of FFM were body mass, and the abdominal and thigh SF; in the regression model, *R* was 0.98 and SEE was 1.1 kg. When used in handball players, the equation of Warner et al. (2004) significantly (*P* < 0.001) overestimated DXA-measured FFM with a MSD = +4,723 g. A reason for such a large discrepancy may be found in the markedly different BC between the two samples, with athletes in the Warner’s study showing a much larger FFM (50 ± 5.9 vs. 42.3 ± 5.59 kg) and %FFM (79.7% vs. 65.2%) vs. the handball players participating in the current study, indicating that the two samples are probably composed by different types of athletes. In fact, the participants recruited by Warner et al. (2004) were women varsity and club athletes practicing different sports (e.g., crew, cross-country, track and field, field hockey, gymnastics, and so on).

Finally, also the predictive equation for FFM of Fornetti et al. (1999) used in this study for cross-validation FFM used DXA as the criterion. The equation was developed in 132 varsity sports athletes (not including participants in handball) showing body mass (74.6 ± 6.7 kg), stature (170.4 ± 8.1 cm), and BMI (22.5 ± 2.5 kg/m^2^) similar to those found in the handball players participating in the current study. The predictors were body mass and stature; the developed model had *R* = 0.961 and SEE = 1.6 kg. The equation of Fornetti et al. (1999) significantly (*P* < 0.001) overestimated FFM vs. DXA FFM with MSD = +2,945 g. Once again, this discrepancy may be explained by the much larger FFM (49 ± 6.0 vs. 42.3 ± 5.59 kg) and %FFM (78.4% vs. 65.2%) in athletes participating in the study of Fornetti et al. (1999) vs. the handball players participating in the current study.

This study has some limitations that should be mentioned. First, this is a single-center study and, therefore, some caution should be applied when expanding our results to the generality of female handball players; caution should also be applied when using the equations developed in our group of handball players to predict BC in elite athletes. In fact, data in the literature (Leyk et al., 2007; Cizmek et al., 2010; Vila et al., 2012; Mala et al., 2015) show that elite handball players tend to be taller (171.3 ± 7.4 cm (Vila et al., 2012) and 176.6 ± 6.5 cm (Mala et al., 2015)) and heavier (67.6 ± 8.1 kg (Vila et al., 2012) and 72.5 ± 8.3 kg (Mala et al., 2015)) and to have lower %FM (19.4 ± 4.5 (Cizmek et al., 2010)) or higher FFM (51.0 ± 2.7 kg (Leyk et al., 2007)) in comparison with players in our sample. Further, the equations presented in this study might show limited accuracy when applied to some playing positions. It is known that different playing positions in handball have different BC (Milanese et al., 2011), as well as physical demands during the play (Michalsik, Aagaard & Madsen, 2015). While in the derivation sample used in this work the number of players per each playing position was similar to that found in a regular handball team, the validation sample contained a limited number of goalkeepers and pivots. Accordingly, some caution should be used when predicting BC for these playing positions with the equations presented herein. Further work carried out in a larger number of players would enable the development of playing-position specific predictive equations for BC in female handball players.

This work, however, has a number of strengths to be highlighted. A main strength of the study is the use of DXA, a reference standard for assessing BC. Moreover, the female handball players participating in this study had demographic characteristics and %BF similar to those shown by a large (*n* = 222) group of players of the Greek league (Bayios et al., 2006) as well as French players (Filaire & Lac, 2000; Garcin et al., 2003) showing that the equations presented herein would be of use in several handball milieus. Finally, the data provided in this study help to fill some important gaps in the literature providing sport-specific anthropometric equations to predict BC in handball with important practical implications for trainers and coaches.

## Conclusion

In conclusion, this work produced a set of validated anthropometric sport-specific predictive equations able to accurately estimate BC components in female handball players. Cross-validation against DXA of several independently developed predictive equations for %FM and FFM in female athletes revealed that they are not accurate when used in female handball players. Sports professionals will therefore benefit from using the predictive equations proposed therein as a rapid and non-invasive tool for assessing and monitoring BC in female handball players.

## Supplemental Information

10.7717/peerj.5913/supp-1 Supplemental Information 1 Raw data.

## Additional Information and Declarations

### Data Availability

The following information was supplied regarding data availability: The raw data are available in a Supplemental File.
